# A methylcellulose/agarose hydrogel as an innovative scaffold for tissue engineering

**DOI:** 10.1039/d2ra04841h

**Published:** 2022-09-21

**Authors:** Beata Niemczyk-Soczynska, Arkadiusz Gradys, Dorota Kolbuk, Anna Krzton-Maziopa, Piotr Rogujski, Luiza Stanaszek, Barbara Lukomska, Pawel Sajkiewicz

**Affiliations:** Institute of Fundamental Technological Research, Polish Academy of Sciences Pawinskiego 5b St., 02-106 Warsaw Poland bniem@ippt.pan.pl; Faculty of Chemistry, Warsaw University of Technology Noakowskiego 3 St. 00-664 Warsaw Poland; NeuroRepair Department, Mossakowski Medical Research Institute, Polish Academy of Sciences 5 Pawinskiego St. 02-106 Warsaw Poland

## Abstract

*In situ* crosslinked materials are the main interests of both scientific and industrial research. Methylcellulose (MC) aqueous solution is one of the representatives that belongs to this family of thermosensitive materials. At room temperature, MC is a liquid whereupon during temperature increase up to 37 °C, it crosslinks physically and turns into a hydrogel. This feature makes it unique, especially for tissue engineering applications. However, the crosslinking rate of MC alone is relatively slow considering tissue engineering expectations. According to these expectations, the crosslinking should take place slowly enough to allow for complete injection and fill the injury avoiding clogging in the needle, and simultanously, it should be sufficiently fast to prevent it from relocation from the lesion. One of the methods to overcome this problem is MC blending with another substance that increases the crosslinking rate of MC. In these studies, we used agarose (AGR). These studies aim to investigate the effect of different AGR amounts on MC crosslinking kinetics, and thermal, viscoelastic, and biological properties. Differential Scanning Calorimetry (DSC) and dynamic mechanical analysis (DMA) measurements proved that AGR addition accelerates the beginning of MC crosslinking. This phenomenon resulted from AGR's greater affinity to water, which is crucial in this particular crosslinking part. *In vitro* tests, carried out using the L929 fibroblast line and mesenchymal stem cells (MSCs), confirmed that most of the hydrogel samples were non-cytotoxic in contact with extracts and directly with cells. Not only does this type of thermosensitive hydrogel system provide excellent mechanical and biological cues but also its stimuli-responsive character provides more novel functionalities for designing innovative scaffold/cell delivery systems for tissue engineering applications.

## Abstract 2

Agarose addition to a methylcellulose (MC) solution accelerates MC thermal crosslinking, enhances mechanical properties, provides an ECM-mimicking environment, and allows homogenous cell infiltration into hydrogel volume.

## Graphical abstract

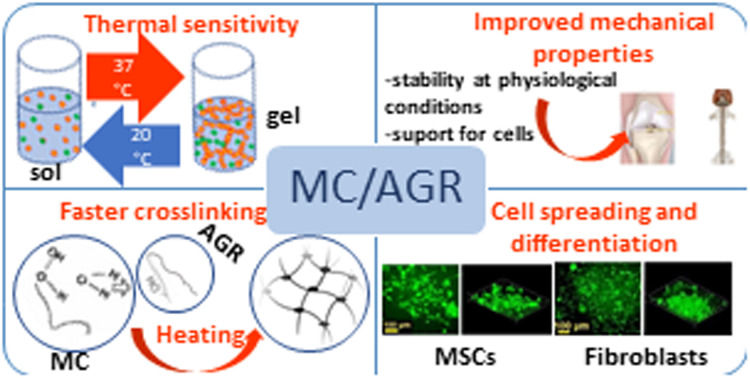

## 1 Introduction

Stimuli-responsive materials have gained attention in many fields of science.^1^ Under appropriate external stimulation, these smart materials might present changes in wettability, swelling behavior, electrical conductivity or show reversible sol–gel transition.^2–5^ Such features have been used in smart hydrogel development, and at the same time, have met the criteria of tissue engineering requirements. Hydrogels are a group of polymers that absorb a large amount of water under particular stimulation in an aqueous environment. They are very attractive from a tissue engineering perspective due to the 3-dimensionality of the hydrogel network and physical resemblance to living tissues.^1^ The application of ultraviolet (UV) radiation, pH, and temperature change are the most common examples of factors inducing crosslinking of stimuli-responsive hydrogels. For instance, UV light might stimulate *in situ* crosslinking of gelatin methacryloyl (GelMA), which serves as a scaffold in many tissue engineering fields. Although UV light can repidly and easily crosslink hydrogels, there is a risk of generating free radicals, resulting in cell mutations and the occurrence of damage in cell DNA.^6^ Another example of stimuli-responsive hydrogels with an application as a scaffold in tissue engineering is sodium alginate, which can be stimulated by pH. Despite its natural origin, good biocompatibility, ease of chemical modification, and attractive release profile of tissue growth factors, it has also shown poor mechanical properties resulting in instability in the physiological environment.^7,8^ Interesting stimuli-responsive materials are natural-based injectable thermosensitive hydrogels, in which crosslinking is stimulated with the change of temperature. Such materials are solutions in ambient conditions, but when temperature increases or decreases, the hydrogel is formed as an effect of physical interactions.^9^ Temperature-responsive systems which crosslink during heating are of particular interest because they might be injected through a syringe to the targeted tissue, where it undergoes *in situ* crosslinking.^10,11^ The following natural-based hydrogels have been used in tissue engineering: Xyloglucan, Chitosan as well as Hyaluronic acid (HA) derivatives. Xyloglucan mixed with poly-d-lysine was used as a scaffold for Central Nervous System (CNS) regeneration and showed good axon response in direct contact.^12^ However, it crosslinks at *ca.* 20 °C, so there is a risk of clogging inside the needle during injection. Chitosan and HA alone are not thermosensitive; therefore, they need to be mixed with other thermosensitive materials. For instance, chitosan/glycerophosphate (GP) composite provides thermal sensitivity and a fast crosslinking rate. Nevertheless, in the end, this system occurred to be cytotoxic.^13^ Whereas, HA was mixed with Pluronic, showing appropriate biocompatibility, release profile, and crosslinking rate as drug delivery systems.^14^ Despite the better functionality of such a blend, it was reported that HA might degrade rapidly in physiological conditions and its mechanical properties might be not sufficient from the perspective of tissue engineering requirements.^15^ Additionally, HA's synthesis is very complicated and expensive, making the material unattractive from an economic point of view.^16^

Obviously, tissue engineering needs a material, which crosslinks at the physiological temperature, is biocompatible, non-toxic for cells, stable under physiological conditions, and displays adequate mechanical properties. High quality and favorable prices of such an approach would also be beneficial. In this respect, an interesting material seems to be methylcellulose aqueous solution (MC), which alone demonstrates thermosensitive behavior. The MC is methylated cellulose, in which some of the hydroxyl groups (–OH) are replaced with the methoxy groups (–OCH_3_). The presence of methoxy groups prevents crystallization, making MC water-soluble.^17^ There were plenty of proposed MC crosslinking mechanisms such as micelle formation,^18^ phase separation,^19^ nucleation and crystallization,^20,21^ or hydrophobic fibril-like domains formation.^22,23^ The last one said that MC undergoes thermal reversible crosslinking throughout temperature increase, resulting in hydrophobic interactions formation. The temperature of crosslinking depends on the degree of substitution with the desired value near 37 °C.^24^ The two-step crosslinking mechanism of MC involves two distinct stages. Briefly, the 1st stage assumes destruction of “water cages” located in the vicinity of nonpolar functional groups. In this instance, nonpolar groups –OCH_3_ are considered.^25^ Generally, this phenomenon is a specific type of “dehydration” leading to –OCH_3_ groups exposure as well as their self-organization, which is terminated with the formation of hydrophobic interactions. The 2nd stage assumes creation of a hydrophobic 3-dimensional network. Recently a new MC crosslinking mechanism regarding fibril formation has been discovered.^20,26^ While temperature increases, MC chains spontaneously self-aggregate and form hydrophobic fibrils. Detailed mechanism of hydrophobic fibril formation has been clarified by Bodvik *et al.*^27^ who indicated that such arrangement of MC chains decreases the energy of the hydrogel system by the highest possible level of minimization of the –OCH_3_ and water interactions.

MC offers many advantages, *i.e.*, it is non-toxic for cells, stable in physiological environments, and cell culture media. MC's mechanical properties might be easily tailored, by adjusting its concentration, to make it suitable for various native tissues.^28^ Contrary to HA, MC is also attractive from an economic point of view. All these features make MC an attractive candidate for tissue engineering applications.^9^ On the other hand, a pure MC solution has some limitations: the first one is a crosslinking rate which is not fast enough for tissue engineering and drug delivery system requirements. The second one is its non-cell-adhesive nature.^18^

To overcome those limitations, MC might be blended with other materials that can act as an MC “crosslinking initiator.” In this instance, the “crosslinking initiation” relies on faster dehydration of water surrounding –OCH_3_ groups. To overcome the issue of MC's poor cell adhesivity, MC might be blended with bioactive proteins (*e.g.*, laminin) or with polymers increasing cell adhesion, *e.g.*, agarose (AGR).^29,30^

The authors' idea is to fabricate such a composite consisting of MC and AGR aqueous solutions. AGR is a purified linear galactan hydrocolloid derived from marine algae that consist of repeating agarobiose disaccharide units.^30^ This polymer also belongs to the thermosensitive hydrogels group, but its crosslinking occurs during cooling below room temperature by the aggregation mechanism, similar to gelatin. The melting point occurs near the physiological temperature; hence it is complicated to use its pure form as an *in situ* crosslinking scaffold for tissue engineering. On the other hand, AGR is a great candidate to obtain thermosensitive blends. According to,^30^ AGR accelerates MC crosslinking by its additional dehydration, leading to improvement of hydrophobic interactions between the polymeric chains that considerably enhances MC's mechanical properties. An additional part of the MC crosslinking mechanism in the MC/AGR system might concern interactions between AGR and MC three-dimensional network.^31^ Since the blending of MC and AGR solutions has been reported in only a few publications,^30^ the cross-linking mechanism of the MC/AGR system is not yet fully known.

Consequently, our studies aimed to investigate and clarify the effect of AGR addition on MC crosslinking's mechanism and kinetics, the mechanical properties of the final hydrogel, and the biological properties at *in vitro* conditions.

## 2 Experimental

### 2.1 Preparation of MC aqueous solution

Methylcellulose (MC, METHOCEL A15LV, Sigma Aldrich) and agarose (SeaPrepR, Lonza) solutions were prepared at various weight concentrations and proportions. At first 1, 2.5, 3, and 5 wt% (w/w) of MC water solutions were prepared and stirred overnight. The final solutions were stored at 4 °C overnight to ensure proper hydration of the polymer.^32^ AGR powder was dispersed in hot water at 80 °C according to,^30^ after which the solution was added to the MC solution in the appropriate ratio. The 1, 2.5, and 5 wt% of MC solutions were mixed with AGR solutions at the wt. ratio of 1 : 0.3; 1 : 0.7 and 1 : 1. The 3 wt% MC was mixed with AGR at the wt. the ratio of 1 : 1.

For purposes of DSC studies, it was necessary to prepare more MC/AGR concentrations, where MC and AGR were mixed in a wt ratio of 1 : 1, at the following concentrations: 1, 1.25, 1.5, 1.75, 2, 2.05, 2.15, 2.25, 2.5, 3, 4, 5 wt%.

### 2.2 DSC

Differential scanning calorimetry measurements were conducted using the Pyris 1 DSC PerkinElmer (USA, Waltham) calorimeter. The measurements were conducted at the same conditions as in our previous studies,^23^*i.e.*, non-isothermally, in the temperature range of −5–100 °C, and at the constant heating–cooling rate of 2 K min^−1^. Each sample was placed into a dedicated hermetic pan to avoid water evaporation. In order to improve the heat flow signal to noise ratio, the sample mass was high (in the range of *ca.* 60–80 mg), the measurements were carried out against a reference water sample of a comparable mass, and the scans were run for 10 cycles and averaged.

The MC thermal effects were normalized to MC mass and subjected to a deconvolution procedure using Nonlinear Least Squares Fitting with an Asymmetric Double Sigmoid Function (ADS) in order to distinguish each peak that comes from a particular crosslinking stage. For fitting Origin2021b software was used.

### 2.3 Rheology

The viscoelastic properties, including storage modulus (*G*′) and loss modulus (*G*′′), were derived from the MC/AGR water solutions. For this purpose, DMA, MCR 301 rheometer (Anton Paar Physica, Germany) was used. Kinetics of crosslinking was studied based on *G*′ measurements of solutions at 37 °C under an oscillatory shear regime. All measurements were carried out in a limited time range. Prior to measurement, the MC/AGR solutions were heated in the DMA setup from 20 °C to 37 °C, at the heating rate of 2 K min^−1^. A cone-plate geometry with a diameter of 39.9 mm, an angle of 0.989°, and truncation of 47 μm was used in every measurement. The geometry was equipped with an extra solvent trap, preventing water evaporation from the solution. A small-amplitude sinusoidal deformation (0.1% strain and 1 Hz frequency) was applied. For the sake of statistics, measurements for each MC/AGR concentration were repeated 3–4 times. The obtained *G*′ values were approximated using the sinusoidal type of function, including Logistic and Biphasic Dose-response functions. Subsequently, the fitting functions were extrapolated to achieve the saturation plateau at a longer time range and then averaged. Two methods have determined the crosslinking kinetics: from the time derivative of the averaged *G*′ and the crosslinking rate, *k*. The first method allowed defining the maximum rate of *G*′ growth, as the hydrogel's maximum crosslinking rate. The d*G*′/d*t* integration allowed to determine the final storage modulus. The second one assumed crosslinking rate *k* determination from the half transition time, at 50% of crosslinking and the beginning of crosslinking, (*t*_onset_). The *t*_onset_, was determined as the tangent intersection to the baseline before starting the thermal effect and the tangent to the rising part of the thermal effect at 37 °C. The transition rate, *k*, was determined as the reciprocal of the time of the half transition with respect to its *t*_onset_.

### 2.4 Biocompatibility analysis

#### 2.4.1 Sample preparation

Before making solutions, MC bulk and AGR bulk were sterilized with ultraviolet light (UV). The UV sterilization took place for 30 min, and during that time, the bulk was slightly shaken every 10 min to assure equal sterilization. Both MC and AGR solutions were prepared in PBS, to avoid hyperosmotic stress, at the same concentrations, described in subsection 2. Additionally, two MC/AGR concentrations, *i.e.*, 3/3 wt% and 5/1.5 wt% were dissolved in DMEM for additional comparison. Before cell seeding, hydrogels were kept for 72 h in the incubator at 37 °C to induce entire crosslinking of the MC/AGR solution.

#### 2.4.2 Fibroblasts culture

*In vitro* tests were carried out with the use of the L929 line of fibroblasts (Sigma Aldrich). Cells were cultivated in 75 cm^2^ flasks containing a medium prepared of High Glucose Dulbecco's Modified Eagle's Medium (DMEM), 10% fetal bovine serum (FBS), and 1% antibiotics. Cells were incubated in a 5% CO_2_ environment at a constant temperature of 37 °C. Harvesting of the cells took place in 70–80% confluent flasks. In the first step cells in phosphate buffer saline (PBS). After that step, 5 ml of 0.05% of trypsin solution was added to the cells and placed in the incubator for a few minutes. Then the flask was tapped delicately in order to detach the cells. After obtaining harvested cells, 10 ml of culture medium was added and centrifuged. The centrifugation was carried out at ambient temperature conditions, at 100 × g, for 5 min. To obtain the required cell density, the pellet was resuspended with a culture medium and then diluted.

### 2.5 Fibroblasts evaluation

#### 2.5.1 Presto blue cytotoxicity tests on extracts

In order to obtain extracts for the cytotoxicity test, 5 samples of each hydrogels type were placed in a 48-well plate. They were immersed in 500 μL of culture medium per well, kept at 37 °C, and gently stirred for 24 h. For reference, along with wells with samples, 5 wells without hydrogel were filled with the medium as well. At the same time L929 cell suspension was seeded into another 48-well plate in the same amount of wells as sample extracts plus control with density 1 × 10^4^ cells per well and put in an incubator for 24 h. The wells filled with DMEM only served as reference. After that time the culture medium from cell-seeded wells was replaced with material extracts and the plate was placed in the incubator for another 24 h.

Presto blue assay assumed reduction of resazurin reagents to a resorufin which was the main cell viability compound. The result of this reduction was changing colour from blue to fluorescent red. This assay allowed for quantitative and visual analysis through absorbance and optical evaluation of fluorescent results of resorufin reduction. After 1 and 3 days of cultivation, DMEM was removed and each well was filled with 180 μL of PBS and 20 μL of Presto blue reagent and then the plate was returned to the incubator for 60 min. This step was completed, and 100 μL from each well was transferred to the 96-well plate. The fluorescence read with excitation/emission 530/620 nm filters was measured with the use of 530/620 nm excitation/emission wavelength by Fluoroscan Ace bnt FL Thermo Fisher Scientific. The results were compared with the Presto Blue fluorescence of blank samples, which did not show metabolic activity, and the control (Tissue Culture Plate TCP), which showed 100% of metabolic activity.

Cellular number was evaluated in studies on extracts. Briefly, the calibration curve was prepared after 1, 2 and 3 days of cell cultivation on TCP based on the known number of cells (TC20 automated cell counter Bio-Rad) and relative fluorescence unit determined (RFU) from Presto Blue for this known number of cells. On day 1 and 3, the cell number of all analyzed hydrogels was evaluated in comparison to TCP.

#### 2.5.2 Fibroblasts morphology

For microscopy analysis cells were seeded on crosslinked hydrogels with the density of 5 × 10^4^ per well in 250 μL of the medium in a 24-well plate. Before analysis, seeded cells were stained with the CellTrace™ Yellow Cell Proliferation Kit (Thermo Fisher Scientific). Briefly, 3 μg of CellTrace dye labelled 10^6^ cells, and the staining was carried out for 20 min. After 1 and 3 days, samples were observed under fluorescence microscopy (Leica AM TIRF MC). Additionally, *Z*-stack images were made to obtain the 3D view of fibroblasts distribution and viability on the hydrogel.

### 2.6 MSCs evaluation

#### 2.6.1 3D culture of hBM-MSCs in MC/AGR hydrogels

Prior to cell seeding 150 μL (single 48-well is covered (6.59 mm^2^) and the height of hydrogel is about 2.36 mm) of each MC/AGR hydrogel blend was inserted into a 24-well Nunc™ Cell-Culture Treated Multidish (Cat. No. 142485; Thermo Fisher Scientific, Waltham, MA, USA) at room temperature (RT), and incubated for 72 hours in a humidified atmosphere at 37 °C and 5% CO_2_ to polymerize. Human Bone-Marrow Derived Mesenchymal Stem/Stromal Cells (hBM-MSCs) were isolated as previously described.^33^ The isolation protocol was approved by the ethical review board of the Medical University of Warsaw, and all samples were processed after informed written consent. 1 × 10^4^ hBM-MSCs in the third passage were seeded per well (V = 1.9 cm^2^) with each MC/AGR hydrogel blend, and cultured in RoosterNourish-MSC-XF medium, composed of RoosterBasal™-MSC (Cat. No. SU-005) supplemented with RoosterBooster™-MSC-XF (Cat. No. SU-016) from RoosterBio, Inc., Frederick, MD, USA, in a humidified atmosphere at 37 °C and 5% CO_2_. Cells seeded in empty wells and cultured in parallel were treated as controls. All experiments were performed in duplicates and repeated at least three times.

#### 2.6.2 Cell viability assessment of hBM-MSCs

LIVE/DEAD™ Viability/Cytotoxicity Kit for Mammalian Cells (Cat. No. L3224; Thermo Fisher Scientific, Waltham, MA, USA) was used to distinguish between live and dead hBM-MSCs, as previously described.^34^ Briefly, 0.5 μL of calcein AM and 2 μL of ethidium homodimer-1 were suspended in 1 ml of sterile PBS, and 200 μL of the solution was added per well, followed by a 20 min incubation at 37 °C in the dark. Stained cells were visualized using a fluorescence microscope Cell Observer SD (Carl Zeiss, Jena, Germany) in *Z*-stack mode, performed in the Laboratory of Advanced Microscopy Techniques, Mossakowski Medical Research Institute, Polish Academy of Sciences.

#### 2.6.3. Morphological analysis of hBM-MSCs

CellTrace™ Yellow Cell Proliferation Kit (Cat. No. C34573; Thermo Fisher Scientific, Waltham, MA, USA) was used to assess the morphology of hBM-MCSs cultured in MC/AGR hydrogel blends. Briefly, 1 × 10^6^ hBM-MSCs in the third passage were labelled with CellTrace™ yellow reagent as per Manufacturer's protocol, and seeded as described in paragraph 2.5.2. Labelled cells were visualized using Leica AM TIRF MC fluorescent microscope (Leica Microsystems, Wetzlar, Germany) in *Z*-stack mode.

### 2.7 Statistical analysis

One-way ANOVA method was used to determine statistical significance between various concentrations of MC/AGR in DMA tests and biological studies. The Tukey test of all pairs determined the statistical significance between individual groups. All statistical analyzes were performed for *p* < 0.05. The statistical analysis was performed using GraphPad Prism 9.4.1 Software. A *p* value of less than 0.05 was considered statistically significant; *p* values are expressed as follows: 0.05 > *p* > 0.01 as*; 0.01 > *p* > 0.001 as**; *p* < 0.001 as***; *p* < 0.0001 as****.

The DMA results and biological studies are presented as the mean value ± SD.

## 3 Results and discussion

### 3.1 DSC

**Fig. 1:**
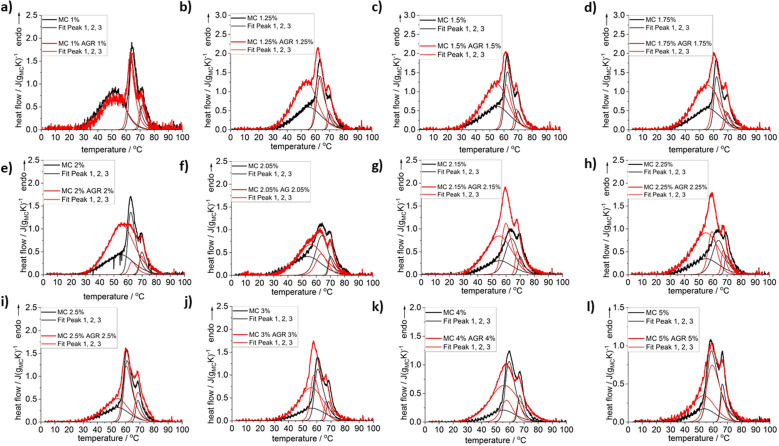
DSC heating scans registered for selected compositions of MC without and with the addition of AGR plus example results of the peak deconvolution. Normalization to MC weight.

The heating scans of MC/AGR concentrations normalized to MC weight after baseline subtraction are presented in Fig. 1. There might be observed several complex over-imposed endothermic effects that come from MC gelation. For all samples, a clear shoulder at low temperatures (LT) and two sharp maxima at medium and high temperatures (MT and HT) are observed. This phenomenon was described in detail in our previous studies.^23^ Briefly, the low-temperature shoulder corresponds to changes in water–water interactions close to polymer chains, due to the water network destruction into smaller clusters. The medium-temperature endotherm (MT) and high-temperature endotherm (HT) correspond directly to the MC crosslinking mechanism, *i.e.*, breaking of “water cages” and subsequent fibril hydrophobic domains formation.^23^

**Fig. 2:**
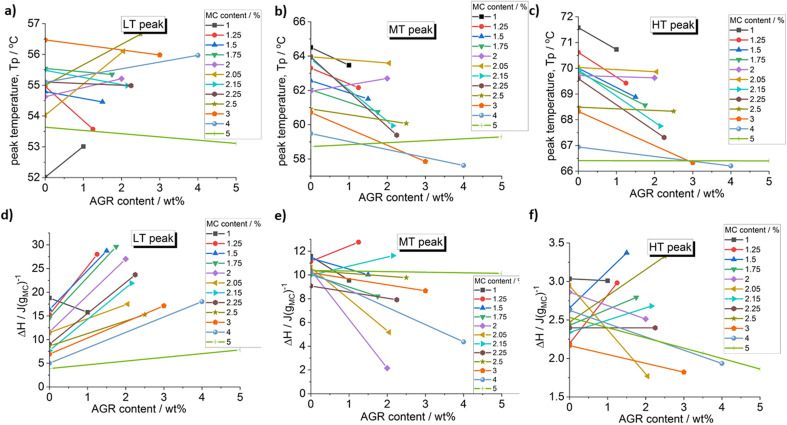
Effect of agarose addition on the peak temperature and the peak heat of the deconvoluted peaks: (a) and (d) LT peak, (b) and (e) MT peak and (c) and (f) HT peak.

In Fig. 1, it may be clearly seen practically for all investigated compositions except for the lowest MC 1 wt%, that addition of agarose increases the heat of thermal effects, especially at low temperature side. To reveal the effect of AGR addition on the individual thermal effects, peak deconvolution analysis using asymmetric double sigmoid function was performed, of which examples are presented in Fig. 1 and the deconvolution results are presented in Fig. 2 as the individual peak's temperature position, TP, and heat, Δ*H*.

From Fig. 2 it may be seen that AGR addition generally strongly increases the heat of the LT peak, corresponding to water–water interactions, while the heats of the MT and HT peaks change variously without clear dependence on MC concentration. A clear shift of the MT and HT peaks to lower temperature is observed, especially, at lower MC concentrations being evidence of crosslinking acceleration after the addition of AGR.

The increase of LT endotherm corresponding to the water molecules interactions, *i.e.*, destruction of the spanning water network into small water clusters,^23^ could be explained by AGRs higher affinity to water.^30^ Since AGR molecules strongly interacts with water molecules, spanning water network destruction is more efficient, which is visible as increase of the LT peak in MC/AGR blends.^30^

The inconclusive trend of MT and HT height after AGR addition could be the result of complex interactions between MC and AGR. Most likely, interactions between AGR and MC chains prevail over MC hydrophobic interactios, decreasing the molecular mobility and resulting MC crosslinking.^28^

### 3.2 Rheology

**Fig. 3:**
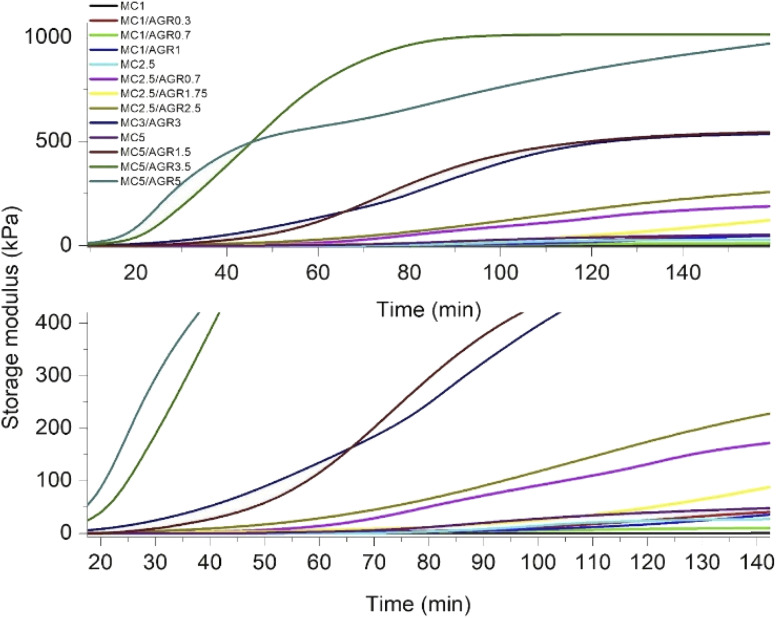
Average *G*′ as a function of time for various concentrations of MC and MC/AGR.

Since *G*′ and *G*′′ curves did not cross, showing the characteristic crosslinking point at a certain time or temperature, as reported by Li *et al.*,^35^ we estimated a crosslinking rate directly by analyzing *G*′ as a time function. All the *G*′ curves of MC and MC/AGR showed a sigmoidal character (Fig. 3). As we presumed, the beginning of crosslinking occurred at a different time among various concentrations, and it was connected with the hydrogel concentration and the amount of AGR in the solution. It is evident from Fig. 3 that irrespective of the MC concentration, the addition of AGR leads to an earlier start and a faster rate of crosslinking which is in line with DSC results. However, in any solution, AGR addition did not accelerate reaching the plateau of *G*′.

**Fig. 4:**
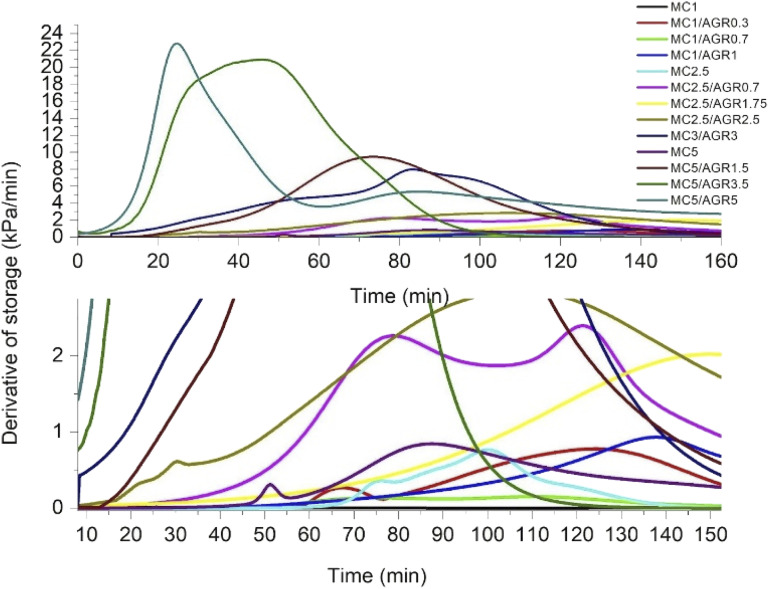
The time derivative of *G*′ for various MC and MC/AGR concentrations.

The *G*′ time derivative analysis clearly indicates that there are at least two local maxima of the crosslinking rate, as it was visible for pure MC (Fig. 4). After mixing 0.7 wt% of AGR with 1 wt% of MC, the 1st maximum of crosslinking rate was observed after 70 minutes. Addition of 1 wt% of AGR to 1 wt% of MC, resulted in a very small maximum after shorter time (*c.a.* 25 min). At this MC concentration, AGR addition significantly affects the beginning of crosslinking (*t*_onset_). The same trend of the first maximum crosslinking rate acceleration is observed at 2.5 wt% MC – mixed with 1.75 wt% of AGR provided the first very small maximum at 30 min while in the case of 2.5 wt% AGR the first crosslinking rate maximum took place after a few minutes. An interesting phenomenon has been observed for 5 wt% of MC, which showed the first maximum of crosslinking rate at 50 min. While after adding 5 wt% AGR, the first maximum was after 90 min, but its intensity was at least 10 fold higher than the first maximum in pure MC. In all cases, the second and further maxima of crosslinking were larger and shifted to the higher time range after AGR addition. Usually, these maxima were stretched in the time range.

Thermal physical crosslinking of MC/AGR is an interesting feature while designing smart *in situ* gelling injectable hydrogels. One of the most important advantages of such an approach is avoiding of chemical modifications and using of toxic crosslinking agents.^36^

DMA results clearly showed that thermal MC crosslinking initiation is strongly dependent on AGR contribution in the MC/AGR solution. The higher the contribution of AGR, the faster the beginning of crosslinking (*t*_onset_) (Fig. 4). As we predicted, AGR plays role of an initiator of MC crosslinking, decreasing *t*_onset_ of crosslinking from dozens to a few minutes. In contrast, AGR did not accelerate the formation of the hydrophobic fibril MC aggregates, resulting in reaching a plateau of *G*′. This means AGR aqueous solution only accelerates the first step of MC crosslinking. The mechanism of MC/AGR crosslinking might be explained in the following manner. At ambient conditions, MC polymeric chains interpenetrate AGR chains, preventing AGR crosslinking. At this temperature, the solution is a sol. The heating of the solution causes MC water cages' destruction, in other words, dehydration. Since AGR has a greater affinity to water, dehydration takes place faster than in pure MC. Martin *et al.*^30^ reported that another reason for faster MC/AGR crosslinking might be the effect of MC and AGR chain interactions. The results of such interactions are forming fibrils through stronger hydrophobic bonds and considerably enhanced mechanical properties of MC.

On the other hand, the second step of MC crosslinking seemed to be prolonged after AGR addition. The reason can be related to the strong interactions between MC and AGR chains resulting in decreased molecular mobility as an effect of the partially crosslinked network formation which slows further hydrophobic interaction formation.^28,37^ However, we should consider that during crosslinking, *G*′ reaches a much higher value in a shorter time after AGR addition (Fig. 3). It could be the effect of additional interactions between MC and AGR polymeric chains.^30,31^ The higher the *G*′ value, the higher the degree of crosslinking, making the whole hydrogel system more stable and provides higher mechanical properties.^38^ Despite the slow changing of *G*′ in time, there is a decreased risk of hydrogel decomposition and displacement from dedicated tissue.

**Fig. 5:**
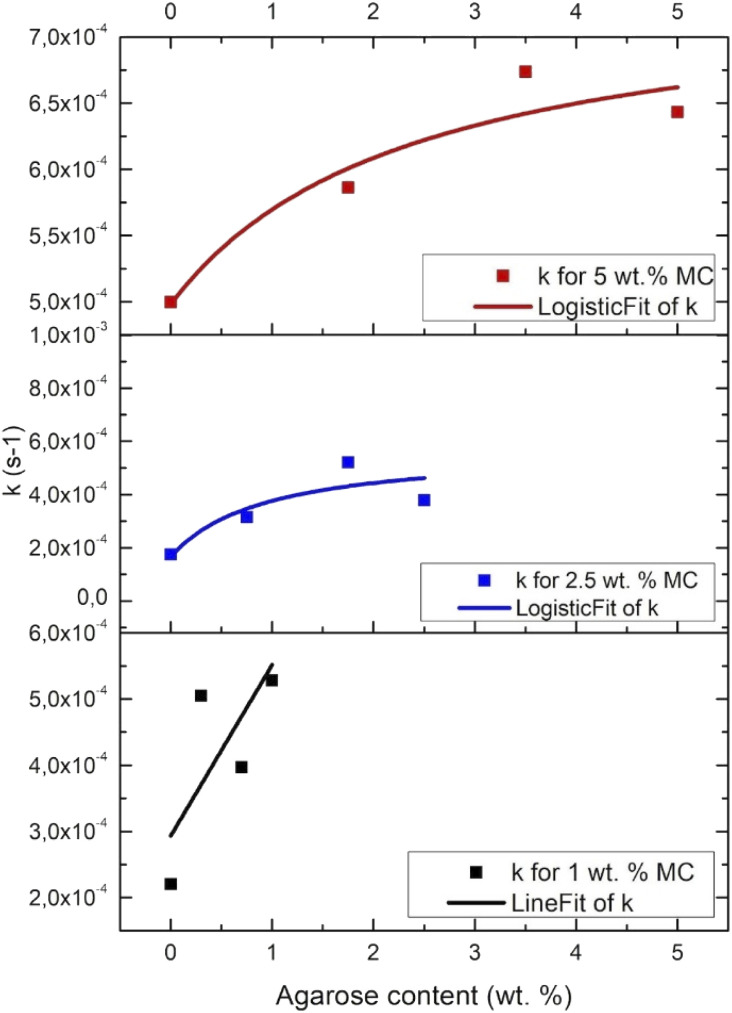
Crosslinking rate (*k*) determined from DMA results, *vs.* the MC/AGR content.

**Fig. 6:**
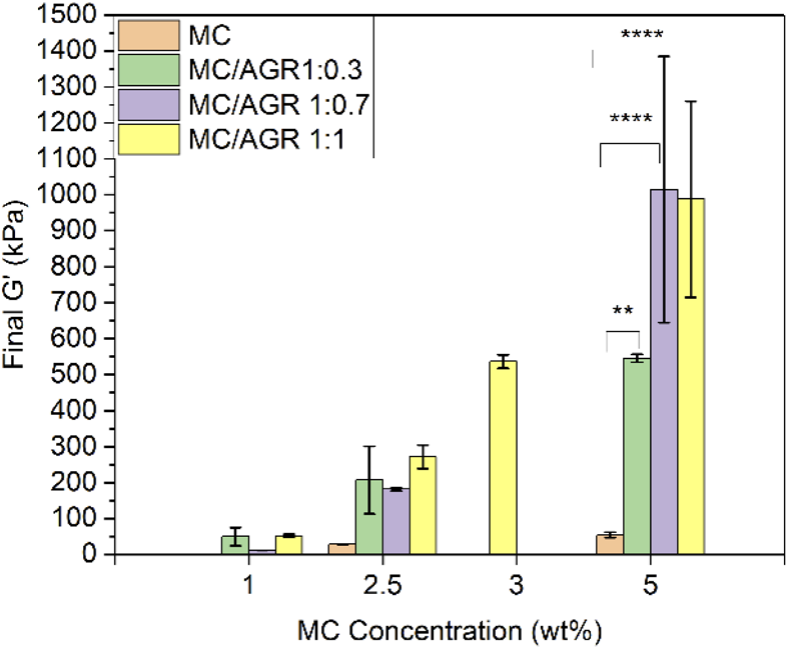
Final *G*′ for various MC and MC/AGR concentrations. Statistical significance **p < 0.001, ****p < 0.0001.

Fig. 5 presents the crosslinking rate, *k*, as derived from the DMA results, as a function of the AGR content. The crosslinking rate increases with the increase of AGR contribution.The final value of *G*′ as a function of all the measured sample concentrations was determined and shown in Fig. 6. The final *G*′ increased with MC concentration and was always higher after AGR addition.

From DMA studies it is evident that small concentrations of these hydrogel systems (1–2.5 wt%) showed, statistically insignificant changes in viscoelastic properties after AGR addition. The values in this range of concentrations correspond to the native human spinal cord. The *G*′ of MC 1/AGR 0.7 is similar to the *G*′ of the human spinal cord which is in the range of 5–42 kPa.^39^ This hydrogel composition might be interesting for CNS tissue engineering considering the perspective of hydrogels' mechanical properties. Above 5 wt% there are visible statistically significant differences in viscoelastic properties after AGR addition, which contribution in the solution increased the final *G*′.

The *G*′ of higher MC/AGR concentrations, *i.e.*, MC 5/AGR 3.5 or MC 5/AGR 5 corresponds to the *G*′ of human articular cartilage.^40^

### 3.3 Biological tests

Small concentrations ∼1 and 2.5 wt% of MC/AGR showed relatively low strength and viscosity after crosslinking, resulting in immediate cells collapsing into the bottom of the well in our preliminary studies. Thus they showed susceptibility to insufficient gelation.^28^ Thus, these concentrations were disregarded in biological tests.

#### 3.3.1 Biocompatibility test

Viability using Presto Blue assay was evaluated on hydrogel extracts of MC 3/AGR 3, MC 5/AGR 1.5, MC 5/AGR 3.5, and MC 5/AGR 5 after 1 and 3 days. Tests were carried out to investigate MC/AGR solutions' relevance in tissue engineering applications.

**Fig. 7:**
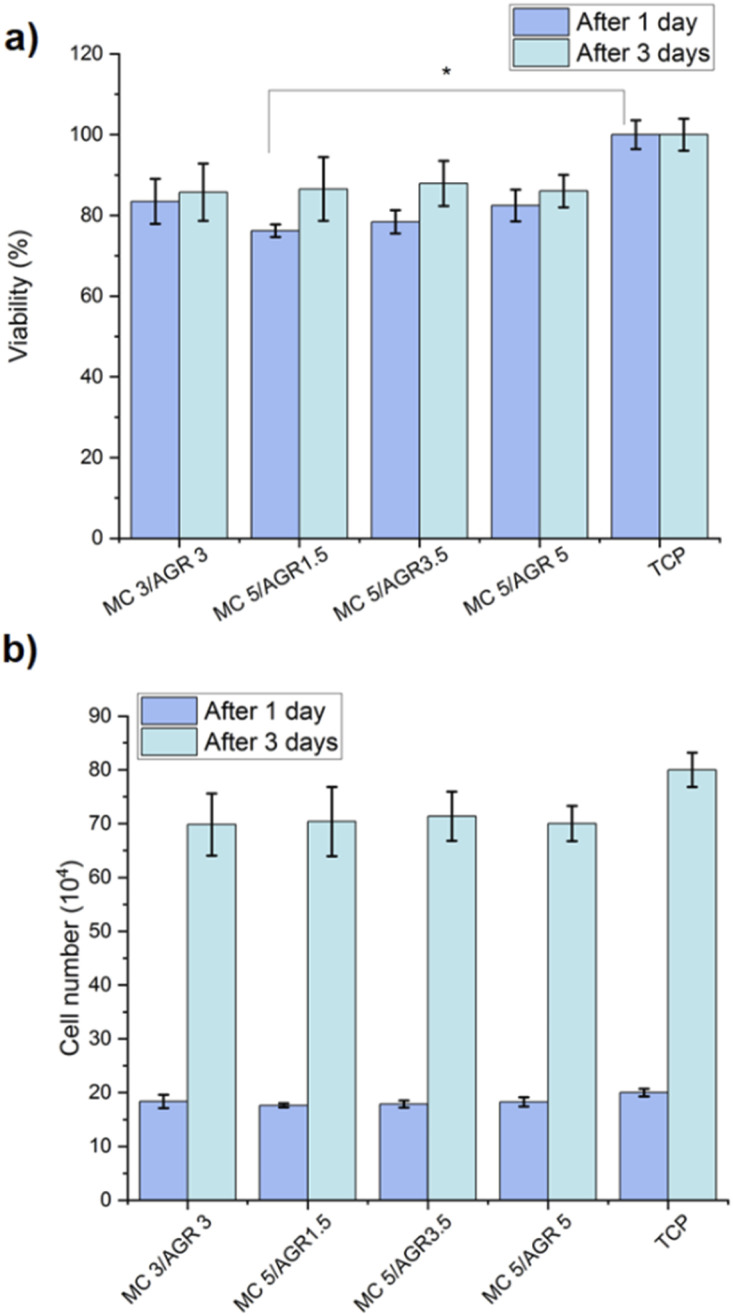
The L929 (a) viability and (b) cell number, determined on MC/AGR hydrogels after 1 and 3 days. Statistical significance: *p < 0.05.

The viability results (Fig. 7a) showed the non-toxic character of the MC/AGR hydrogel. There was slightly lower viability for hydrogel samples in comparison to the control (TCP). Yet all of these samples accomplished values ≥70%, which is considered non-toxic to the living cells according to the standard of ISO 10993-5. Most likely the cells could be covered with thick layer of diluted hydrogel. Szot *et al.* reported increased hydrogels layer thickness could provide hypoxia and limited nutrient diffusion.^41^ According to this, the balance between the migration of cellular waste products and the supply of fresh supplements/proteins from the medium was not maintained, resulting in a reduced viability value after 1 day. However, cellular viability increased after 3 days of cultivation due to fresh media additives.

Additionally, cell number after 1 and 3 days during *in vitro* study on extracts was estimated based on calibration curve for known number of cells (determined by flow cytometry Bio-Rad) and relative fluorescence unit (determined from Presto Blue) (Fig. 7b). The results showed the differences of cell number between studied MC/AGR concentrations were statistically insignificant.

#### 3.3.2 Fibroblasts morphological observation

**Fig. 8:**
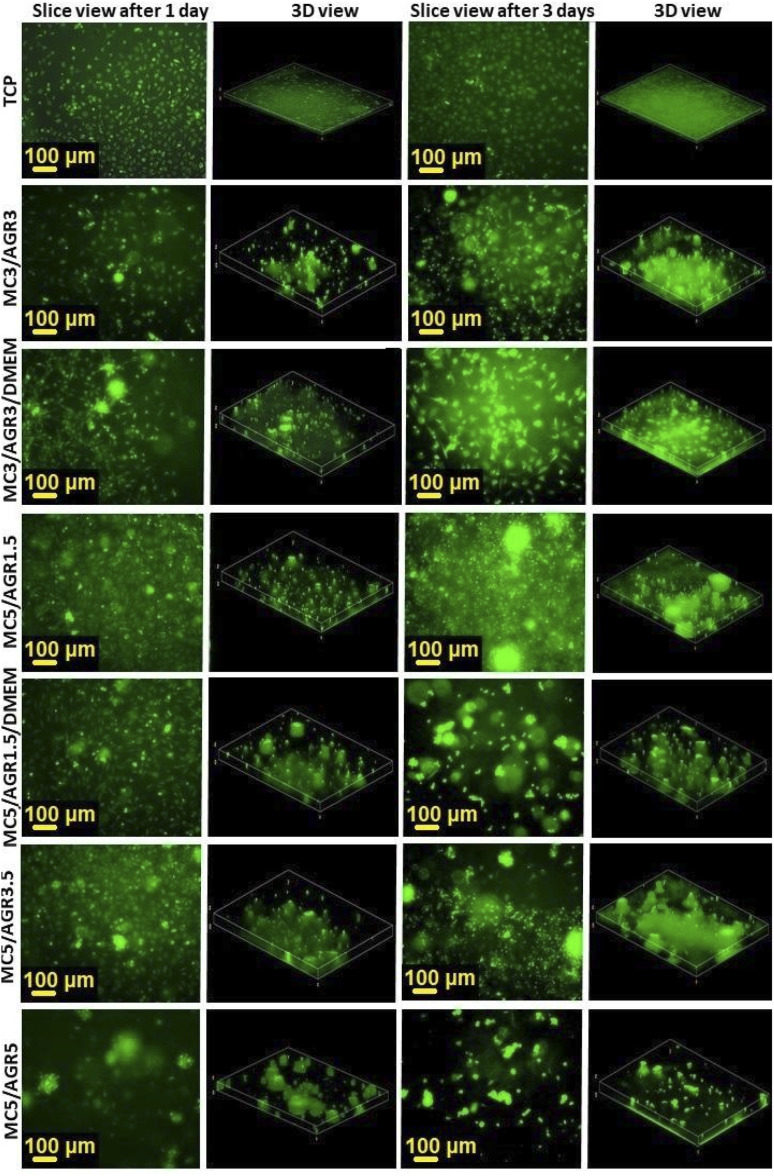
Fibroblasts distribution in 3D cultures by an FM after 1 and 3 days. The slice views (the bottom of the well) and 3D views show various distributions in 3D culture depending on MC/AGR concentration.

Fig. 8 represents fluorescence microscope imaging (FM) of the cells on MC/AGR hydrogels with various MC and AGR ratios. The images presented fibroblasts' distribution in the volume of hydrogel in relation to the control in 2D hydrogel-surface culture and 3D culture. The 3D view not only allows precise observation of cell penetration into the volume of hydrogel but also mimics *in vivo* cell growth. To evaluate exact cell distribution and investigate whether cells infiltrated the entire hydrogel volume, 3D views based on *Z*-stack images of hydrogels have been prepared (Fig. 8). The 3 D views were compared with images taken from the bottom (one slice). All of the tested hydrogels showed good viability and cells were distributed in hydrogel rather homogeneously. However, for other samples, there were visible some cellular aggregates which had round shapes. It is especially visible for MC5/AGR1.5/DMEM and MC 5/AGR5. This is most likely an effect of insufficient oxygen availability in those parts of hydrogel resulting in cell death or uneven crosslinking rate in these particular samples.

The best fibroblasts distribution in the hydrogel was observed for MC3/AGR3/DMEM, MC5/AGR1.5, and MC5/AGR3.5. These three samples also showed the significant density and viability of the cells in comparison to the control.

#### 3.3.3 Viability of hBM-MSCs cultured in methylcellulose/agarose hydrogels

**Fig. 9:**
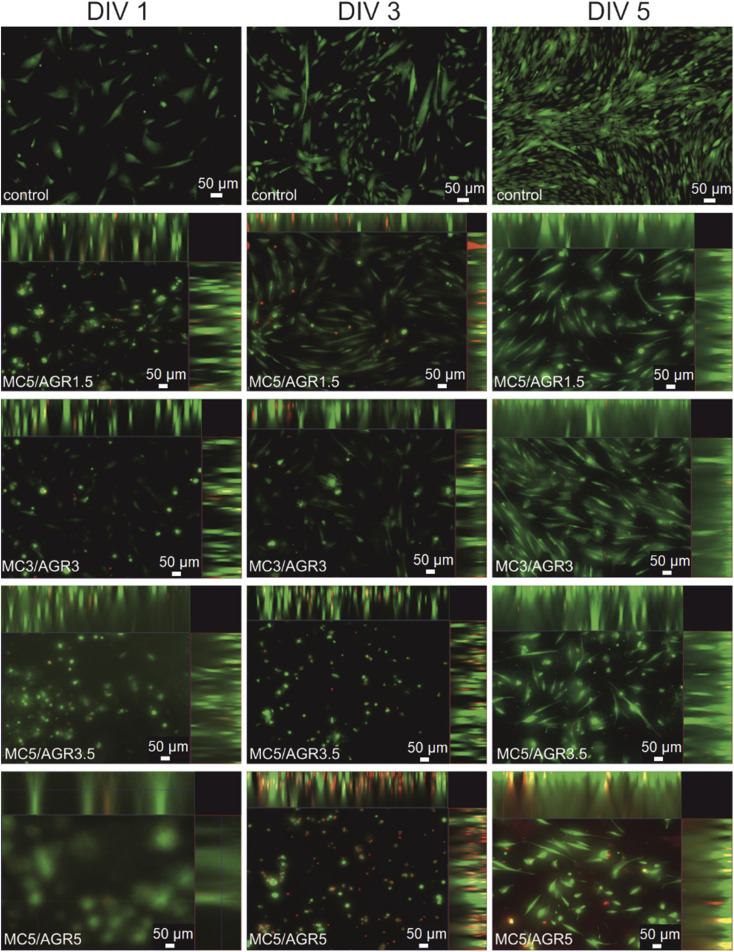
Representative pictures, rendered by maximum intensity projection (MIP) in orthogonal projection, of hBM-MSCs seeded and cultured for 5 days on the following hydrogel combinations: MC5/AGR1.5, MC3/AGR3, MC5/AGR3.5, and MC5/AGR5.

To assess the viability of hBM-MSCs cultured in methylcellulose/agarose hydrogels cells were stained with calcein AM and ethidium homodimer-1 (EthD-1). In living cells, calcein AM is converted into green-fluorescent calcein, whereas in dead cells EthD-1 binds to DNA and emits red fluorescence. Stainings and microscopic analyses were performed at three-time points: one, three, and five days after seeding cells in MC/AGR hydrogels which polymerized for 72 hours. All compositions of hydrogels were non-toxic for hBM-MSCs (Fig. 9). The cells encapsulated in hydrogels remained alive throughout the whole time of culture. The highest number of dead cells was visible in the MC5/AGR5 combination suggesting its negative influence on cell viability. It might be related to relatively high viscosity of hydrogel and decreased gas and/or nutrient exchange. In the case of MC5/AGR1.5 and MC3/AGR3 combinations, some of the cells migrated to the bottom of the cells already after the 1st day of culture probably due to appropriate viscosity of the hydrogel. On the other hand in the MC5/AGR3.5 and MC5/AGR5 time of migration throughout the hydrogel was longer – most of the cells migrated after 5 days of culture. Therefore, depending on the therapeutic approach one could achieve appropriate conditions for the cell migration by manipulation of hydrogel composition.

#### 3.3.4 MSCs morphological observations

**Fig. 10:**
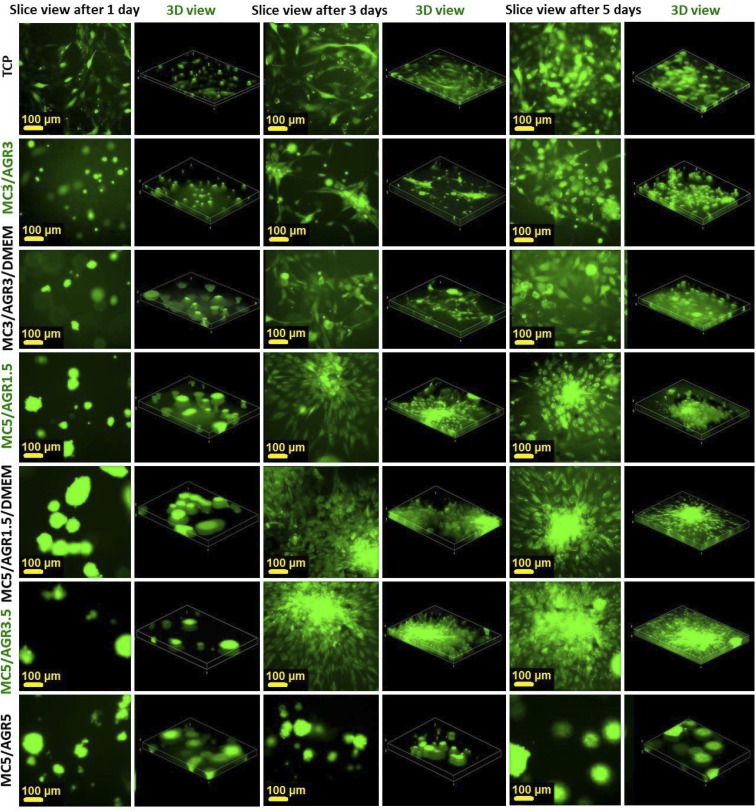
Cell distribution in 3D cultures by an FM after 1, 3, and 5 days. The slice views and 3-D views show cell distributions in 3D culture depending on MC/AGR concentration.

Morphological MSCs distribution and their penetration into the hydrogel volume are illustrated in Fig. 10, where 2D and 3D views obtained *via* FM are shown. It is visible that cells were suspended in all volumes of the hydrogel after one day. However, the FM images of MC5/AGR5 were different from other samples. It showed a small number of cells formed in aggregates that have round shapes in comparison to the control.

For other samples, after 3 and 5 days cells adhered and proliferated on the bottom of the wells, showing adequate morphology and distribution in comparison to TCP control. The adequate MSCs distribution in the hydrogel was observed for most of the samples. The shape of the cell depends on its origin, environment and time of cultivation.^42^ According to the image of viability evaluated by live dead test (Fig. 9), after 5 days most of the MSC probably migrated to the bottom of the well which is more stiff than hydrogel. That is the reason why cells were more spread/elongated and less spherical. This effect was not observed for L929 which indicates lower cellular volume and probably does not reach well bottom (Fig. 8).^42^ Additionally, interactions between the cell and hydrogel depend on mechanical properties of the hydrogel. According to literature, the difference in the stiffness of hydrogels determine cellular area.^43^ Authors present that the area of cells seeded on stiffer hydrogel was statistically significantly higher than on soft hydrogel.

The cell migration through the volume of the hydrogel might be beneficial from the perspective of cell delivery systems. It seems that investigated hydrogels (except for MC5/AGR5) can provide a supportive environment, mechanically protecting the cells during the transplantation procedure. Additionally, the structure of the hydrogel allows for cell migration within the scaffold and thus enables the settlement of transplanted cells in the host tissue. It is especially visible after one day of cell culturing (Fig. 10) where cells embedded in hydrogel showed spherical morphology. According to Kim *et al.*^38^ this morphology is beneficial for embedding cells in hydrogel matrix approaching as a potential injectable cell delivery system. Thus, most of the MC/AGR concentrations could be used as MSCs delivery systems that provide faster regeneration of injured tissues.

## 4 Conclusions

Since MC crosslinking, especially its first step, is mostly dependent on water cages forming/breaking mechanism, the addition of agarose is essential here. AGR has greater affinity to water and effectively uptakes water molecules from MC solution, resulting in faster MC crosslinking.

Our DSC and DMA results that are mutually consistent, clearly demonstrate the addition of AGR promotes MC crosslinking by effective uptaking of water molecules from MC solution, resulting in an easier hydrophobic interaction formation.

In the case of DSC this promotion of MC crosslinking is visible for the low temperature effect as an increase in the LT peak area and a shift of the MT and HT effects towards lower temperatures (times).

Isothermal measurements of time dependence of *G*′ indicated that *t*_onset_ of crosslinking is faster with the increase of AGR contribution in the solution. The AGR has a significant influence on MC crosslinking initiation, but at the same time, does not play a role in further steps of MC crosslinking. Additionally, the presence of AGR in the system resulted in improved mechanical properties, *i.e.*, the final *G*′ value of the MC hydrogel systems which was most prominent for small concentrations. On the one hand, the final value of *G*′ small MC/AGR concentrations implies that they might be useful as scaffolds for CNS tissue engineering from the mechanical point of view. But on the other hand, *in vitro* tests verified that small MC/AGR concentrations do not provide adequate support for cells as scaffolds or cell delivery systems. Therefore they do not meet the expectations of tissue engineering.

The appropriate AGR contribution in the MC/AGR hydrogel systems of higher concentration, not only did provide adequate crosslinking rate and enhanced mechanical properties, but also influenced good cellular response *in vitro* and showed non-toxic character. However, the desired mechanical properties from the perspective of certain native tissues do not guarantee expected *in vitro* results. For instance, Bonetti *et al.*,^44^ crosslinked chemically MC to increase its stiffness and cellular response but *in vitro* tests did not show significant differences in adhesion and proliferation of L929 fibroblasts seeded on chemically crosslinked and non-crosslinked MC. In our studies, MC5/AGR5 is supposed to be a perfect biomaterial, especially for cellular support from the mechanical perspective. However, the sample was too viscous, which resulted in disturbing the balance between oxygen/nutrients delivery and draining of cellular metabolites.

On the other hand, the two hydrogel systems of MC3/AGR3 and MC5/AGR3.5 have decent mechanical and biological properties showing the best potential as a smart injectable scaffold/cell delivery system for tissue engineering. Therefore, the comprehensive understanding of MC/AGR properties, *i.e.*, MC gelation and its kinetic after AGR addition, mechanical and biological properties, is essential to materials' proper design for future *in vivo* studies.

## Author contributions

B·N·S., D. K. and P. S. conceived the project. B. N. S. optimized and developed the hydrogels. B. N. S. and A. K. K. performed and analysed the DMA experiments. B. N. S. and A. G. performed DSC experiments. A. G. analysed DSC experiments. D. K. performed *in vitro* tests on fibroblasts. B. N. S. and D. K. analysed and performed morphological observations of fibroblasts and mesenchymal stem cells. P. R., L. S. and B. L. performed *in vitro* tests on and mesenchymal stem cells, analyzed and performed morphological observations. B. N. S, A. G., D. K., A. K·K., P. R., L. S., B. L. and P. S. wrote the manuscript. B. N. S. provided financial support.

## Conflicts of interest

There are no conflicts to declare.

## Supplementary Material
